# Supplementary material for: A Comparative Study of HA/DBM Compounds Derived from Bovine and Porcine for Bone Regeneration
Source: J Funct Biomater. 2023 Aug 24;14(9):439. doi: 10.3390/jfb14090439 (PMC10532284; doi:10.3390/jfb14090439)
Supplement: Supplementary file 1 [file jfb-14-00439-s001.zip › jfb-2529283-supplementary.pdf]

Support Information (SI-1): Hydroxyapatite Crystallinity

The JCPDS 9-432 standard was used for hydroxyapatite (HA) characterization through X-ray diffraction analysis [45]. The characterization results of HA extracted from porcine (P) and bovine (B) sources, as well as commercial HA-Ctrl, are illustrated in Figure S1.

The standard diffractogram of apatite (code: 98-026-1063) was well matched by the three HA spectra, confirming the presence of crystalline HA in all of the samples. The Ca/P ratio for Ha-Ctrl was 1.66, whereas for bovine and porcine it measured at 1.68. Notable differences in crystallinity were observed between the animal-derived samples and the commercial HA, based on the peak intensities and shapes. Specifically, broader peaks with less sharp bands were exhibited by the porcine HA compared with the commercial and bovine HA, representing lower degree of crystallinity.

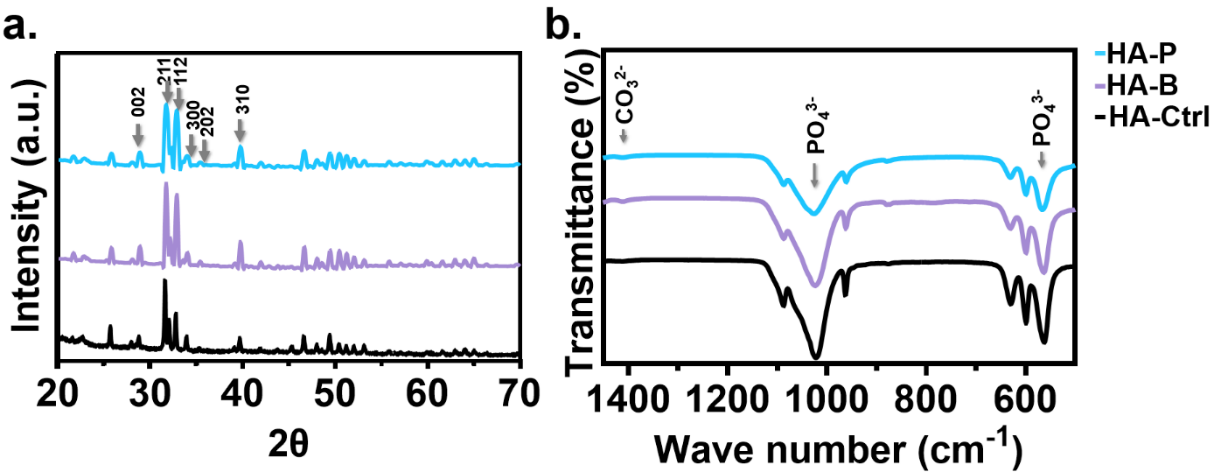

Support Information Figure S1. Characterization of HA extracted from porcine (P) and bovine (B) sources and commercial HA-Ctrl. (a) X-ray diffraction pattern of the extracted HA and their lattice planes. (b) Selected FTIR spectra of the extracted HA indicate the characteristic peaks corresponding to the vibrations of the phosphate (PO_4^{3-}) groups.

Support Information (SI-2): In Vitro Biodegradation Profiles

In this study, we conducted a thorough evaluation and comparison of the in vitro biodegradation profiles of four HA/DBM compounds: 50/50P (blue), 50/50B (red), 60/40P (yellow), and 60/40B (green). The evaluation was carried out at three distinct time points: 0, 1, and 7 days (**SI- Figure S2**).

To conduct the degradation test, we followed the ASTM F1634-95 standard as a reference, with modifications tailored to the type of material and the available resources [34]. Initially, the mixtures were loaded into a 5cc syringe, with the tip pre-cut to extrude 2cc cylinders. The initial weight of these cylinders was carefully measured. Subsequently, the samples were dried at 90°C for 3 hours and then immersed in a pH 7 buffer at a temperature of 30°C for 7 days.

During the evaluation, we recorded the degradation of the samples on days 0, 1, and 7 to observe the trend of weight loss and to calculate the degradation percentage. Additionally, a qualitative image evaluation before drying was performed to visually inspect the HA/DBM compounds under study. The objective was to assess their structural integrity and any observable changes or degradation patterns present at the specified time points.

For further analysis of the biodegradation profiles, we employed a quantitative approach, generating graphs that depicted the degradation rates and patterns of the compounds over the 0, 1, and 7-day time intervals. The weight loss was recorded by removing the samples from the solution and drying them under a vacuum for 3 hours. We utilized the obtained data to calculate the degradation percentage using the relevant equation.

The combination of both qualitative and quantitative evaluations provided comprehensive insights into the degradation behavior of HA/DBM compounds. Notably, our study revealed significant differences in the degradation rates between different animal species and concentrations. Specifically, the degradation rate in porcine was found to be higher than that in bovine.

$$\% \, Degradation = \frac{M_0 - M_T}{M_0} \times 100 \, \%$$

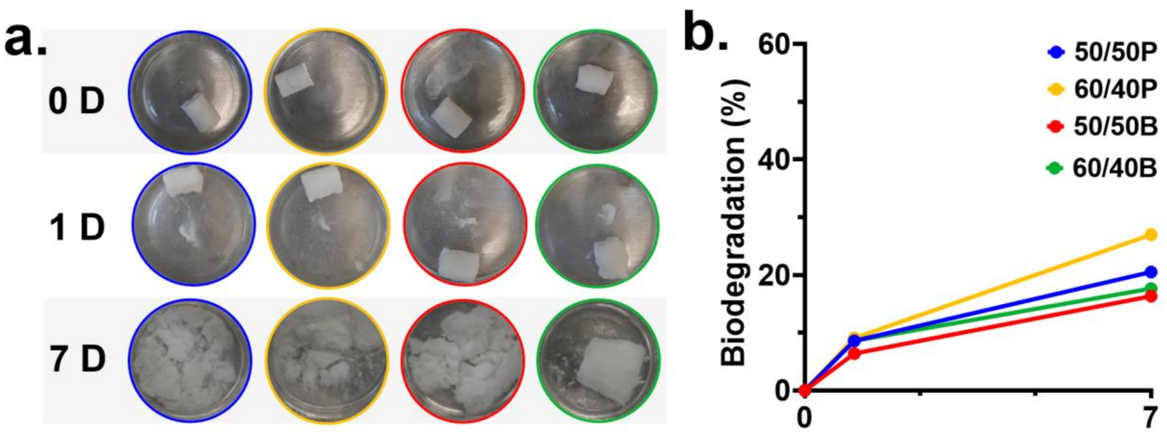

Support Information Figure S2. Evaluation and comparison of the in vitro biodegradation profiles of HA/DBM compounds 50/50P (blue), 50/50B (red), 60/40P (yellow), and 60/40B (green) compounds at three different time points of 0, 1 and 7 days. (a) Qualitative image evaluation before drying. (b) Quantitative graphic representation.

Support Information (SI-3): Inflammatory Response Evaluation and Osteoblastic Differentiation

With the purpose of investigating the inflammatory response generated between bovine and porcine HA/DBM compounds, a complement of the inflammatory response and bone differentiation was carried out using bone-marrow-derived mesenchymal stem cells (BMSCs). Only 50/50P, 50/50B, and commercial DBM (Puros®

Cancellous Particulate Allograft) were evaluated as a complementary material to the obtained results. BMSCs was assessed in cells cultured up to passage 3, following the seller's guidelines. Compounds were added to 24-well plates containing mesenchymal stem cell basal medium (ATCC-LGC standards, Teddington, U.K.). The wells were incubated overnight at 37°C with 5% CO_2, and subsequently replaced with complete medium. The BMSCs were then seeded in a complete medium at a density of 5×10^4 cells/cm^2 in 3 mL of medium per well. A positive control of differentiated cells was obtained by culturing the BMSCs with the osteocyte differentiation toolkit (ATCC PCS-500-052). This complete differentiation medium was replaced at days 2, 3, 7, and 14 of culture. The production of TNF-α and IL-6 by the BMSCs was measured in the cell supernatants from day 3 using ELISA immunoassays (R&D Systems) (**SI- Figure S3**), and gene expression by RT-qPCR evaluations (**SI- Figure S4**) were performed directly on the well plates at days 7 and 14. The in vitro evaluation was performed in triplicate. The selected genes for bone differentiation and generation were *Runt-related transcription factor 2* (*RUNX2*), *Collagen I* (*COLI*), and *Alkaline Phosphatase* (*ALP*), with *GAPDH* used as the internal control. RNA extraction and gene expression assays were performed using the Qiagen RNeasy Mini Kit according to the manufacturer's instructions. The cells were lysed using 600 µL of buffer RTL. The amount and quality of RNA were evaluated using a NanoDrop from Thermo Fisher Scientific. The RT2 First Strand Kit from Qiagen in Hilden, Germany, was used to synthesize cDNA following the manufacturer's instructions, using 100 ng of RNA. RT-qPCR was conducted on a Bio-Rad CFX96 thermocycler, using an iTaq Universal SYBR Green Supermix from Bio-Rad. Gene expression was normalized using the comparative Ct (ΔΔCt) method. Each experimental group and gene primer were analyzed in triplicate.

Chronic inflammation is one of the most evident and common pathological settings that often leads to deregulated bone remodeling. In this study, TNF-α levels were below the detection limit (lower than the negative control). However, IL-6 was detected, and porcine compounds exhibited lower values compared with bovine sources (**SI- Figure S3**).

Similarly, the porcine compounds of HA/DBM demonstrated early values of bone differentiation markers on day 7, whereas the bovine compounds exhibited higher responses at 14 days, although not exceeding those of the porcine compounds on day 7 (**SI- Figure S4**).

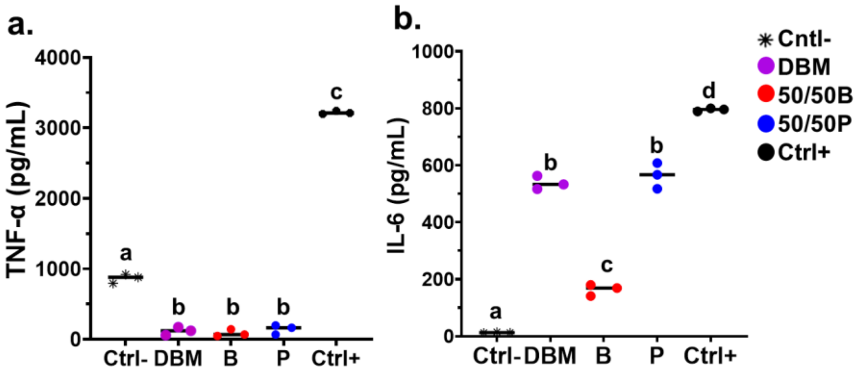

Support Information Figure S3. In vitro study of porcine and bovine-derived bone compound. (a) IL-6 and (b) TNF-α expression after 3 days.

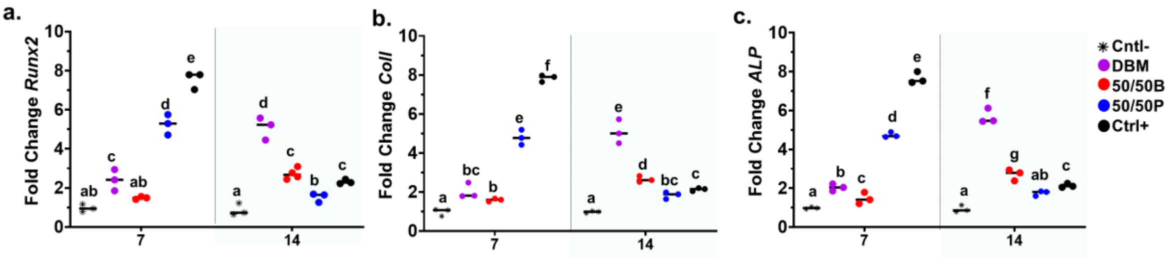

Support Information Figure S4. In vitro study of porcine and bovine-derived bone compound expression of *RUNX2, COLI,* and *ALP* osteogenic proteins.

Support Information (SI-4): Regeneration Treated and Control Defects

The in vivo model was evaluated at 3 and 6 months, comparing the four compounds (50/50P, 60/40P, 50/50B, and 60/40B) and their clinical reference DBM (Puros® Cancellous Particulate Allograft). Forty-four rats were included in the entire experiment and distributed randomly between groups according to (**SI- Figure S5a**). The ANOVA analysis was performed for the treated and natural healing groups on the different variables, including tissue thickness regeneration (%), regenerated bone area (%), regenerated collagen area (%), and blood vessel area (%).

The regeneration thickness (%) showed that the defects treated with HA/DBM had significant superior differences from the controls, except for 3 months of 60/40P, 50/50B, and 60/40B, where it was statistically the same. In the regenerated bone area, at 3 months, only the 50/50P was significantly better than its control. Compared with their controls, all groups performed significantly better after 6 months. The regenerated collagen area at 3 months was the same between the treated and control groups, except 60/40B, where the amount of collagen was higher in the control. At 6 months, the 50/50P control was significantly higher than the treated group. In the blood vessel area (%) at 3 months, 50/50P was significantly higher in the control than in the treatment.

a. Experimental Design

	DBM	50/50P	60/40P	50/50C	60/40C
3 Months	4	5	4	5	4
6 Months	5	4	4	5	4
Total	9	9	8	10	8

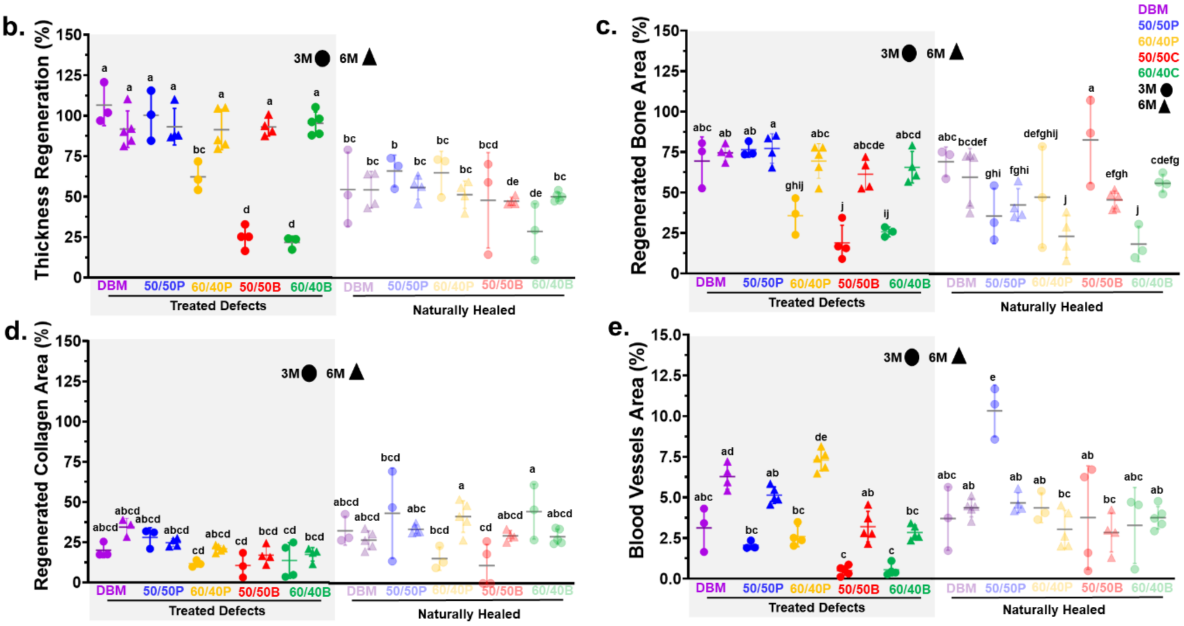

Support Information Figure S5. Treated defects, control defects (natural healing), and the difference between the medians per group for different variables. a. Experimental Design. b. Tissue thickness Regeneration (%) c. Regenerated Bone Area (%). d. Regenerated Collagen Area (%) e. Blood vessels' area (%). Means with different letters represent that significantly different from each other ($p \leq 0.05$).

Support Information (SI-5): Bone Tissue Regeneration Methodology

ImageJ software (NIH, Bethesda, MD, USA) was used to process and calibrate all of the images. The tissue thickness regeneration percentage was measured as the new tissue's average thickness ratio to the original calvarial thickness of the defect [42]. After, four random measurements of the regenerated tissue thickness were averaged, divided by the average of the non-perforated bone calvarial, which served as a reference of the original tissue thickness; the result was multiplied by 100% (**SI- Figure S6a**). The specific percentage of bone (**SI-Figure S6b**) and collagenous tissues (**SI-Figure S6c**) was manually traced and divided by the total regenerated area [43]. This total regenerated area was calculated as the ratio of newly formed tissue (mm^2) over the total defect area (mm^2) [49].

The blood vessels' area (%) was chosen using a histogram and quantified by measuring the ratio between the blood vessels' area (mm^2) (**SI-Figure S6d)** and the total regenerated area (mm^2) [44]. **SI-Figure S6e** shows the different evaluated tissue: bone, and collagen . Finally, **SI-Figure S6f** shows a 15-day postoperative evaluation after surgery; the rats' weight and temperature were evaluated.

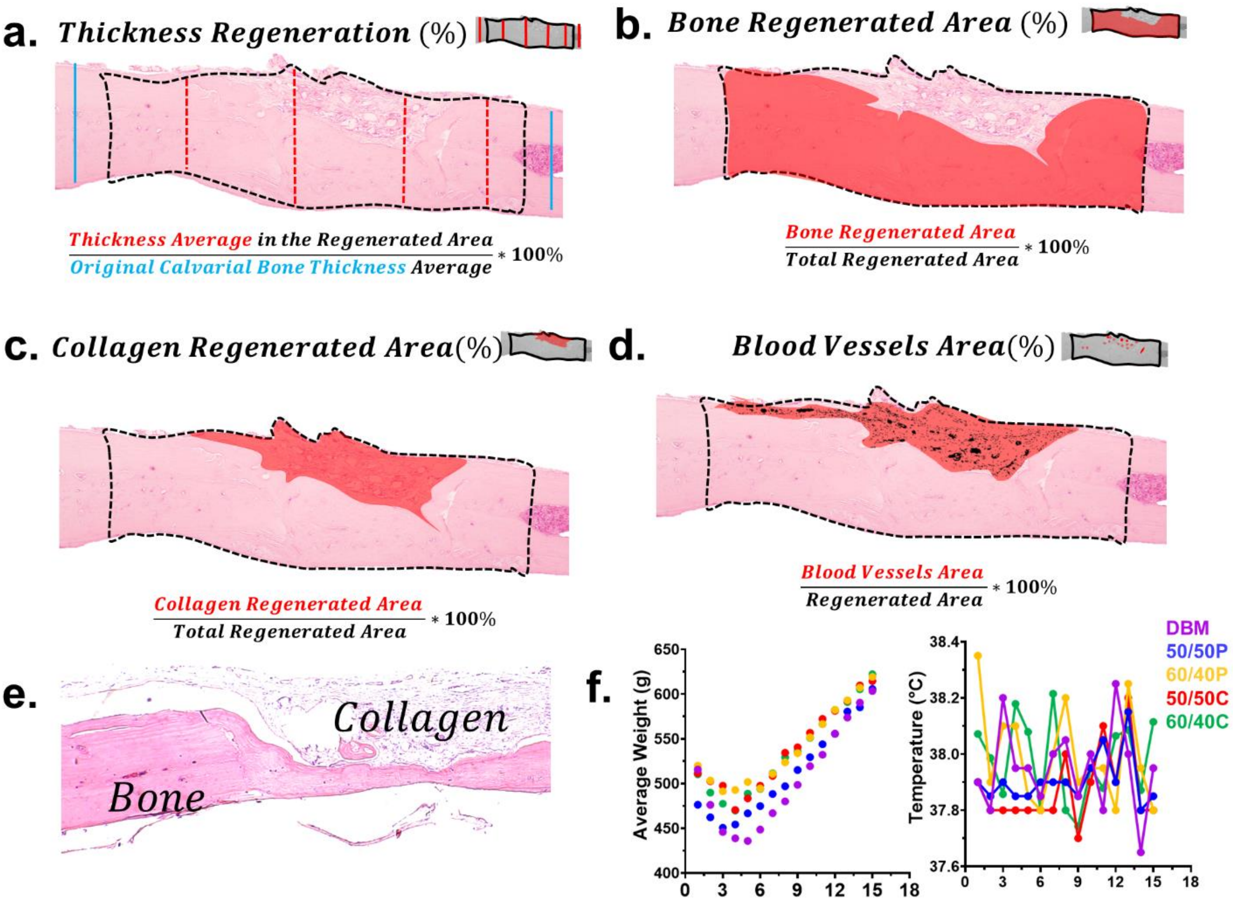

Support Information Figure S6. Methodology for quantitative evaluation of regeneration and inflammation. (a) Tissue thickness regeneration (%). (b) Regenerated bone area (%). (c) Regenerated collagen area (%). (d) Blood vessels' area (%). (e) Different evaluated tissue, bone, and collagen. (f) The 15-day postoperative evaluation, rats' weight, and temperature after surgery.
